# Regulation of human bone marrow stromal cell proliferation and differentiation capacity by glucocorticoid receptor and AP-1 crosstalk

**DOI:** 10.1002/jbmr.120

**Published:** 2010-10

**Authors:** Iván Cárcamo-Orive, Ainhoa Gaztelumendi, Jesús Delgado, Naiara Tejados, Akaitz Dorronsoro, Jon Fernández-Rueda, Daniel J Pennington, César Trigueros

**Affiliations:** 1 Fundación Inbiomed, Foundation for Stem Cell Research, Mesenchymal and Hematopoietic Stem Cell DepartmentPaseo Mikeletegi, San Sebastián. Spain; 2 Institute of Cell and Molecular Science, Barts and The London School of Medicine and Dentistry, Queen Mary London UniversityLondon, UK

**Keywords:** osteoblasts and stem cells, bone, adipose, corticosteroid osteoporosis

## Abstract

Although marrow adipocytes and osteoblasts derive from a common bone marrow stromal cells (BMSCs), the mechanisms that underlie osteoporosis-associated bone loss and marrow adipogenesis during prolonged steroid treatment are unclear. We show in human BMSCs (hBMSCs) that glucocorticoid receptor (GR) signaling in response to high concentrations of glucocorticoid (GC) supports adipogenesis but inhibits osteogenesis by reducing c-Jun expression and hBMSC proliferation. Conversely, significantly lower concentrations of GC, which permit hBMSC proliferation, are necessary for normal bone mineralization. In contrast, platelet-derived growth factor (PDGF) signaling increases both JNK/c-Jun activity and hBMSC expansion, favoring osteogenic differentiation instead of adipogenesis. Indeed, PDGF antagonizes the proadipogenic qualities of GC/GR signaling. Thus our results reveal a novel c-Jun-centered regulatory network of signaling pathways in differentiating hBMSCs that controls the proliferation-dependent balance between osteogenesis and adipogenesis.

## Introduction

Several types of cells constitute bone: osteoblasts (bone-forming cells), osteoclasts (bone-reabsorbing cells) and osteocytes, which are mature osteoblasts that become trapped and surrounded by self-generated bone matrix. Bone tissue is constantly reabsorbed by osteoclasts and replaced by osteoblasts, a process known as *bone remodeling* that is under paracrine control to maintain bone integrity in healthy individuals. It is believed that precursors of osteoclasts share properties with the monocyte-macrophage cell lineage, suggesting a hematopoietic origin.^1^ Conversely, osteoblasts, and therefore osteocytes, descend from bone marrow stromal cell lineages (BMSCs). BMSCs serve as precursors for various mesodermal tissues, including cartilage, adipose, connective, and the aforementioned osseous tissues.^2^, ^3^

Osteoporosis is a debilitating condition characterized by low bone mass and increased bone fragility. This loss of bone appears to coincide with declining numbers of osteoblasts and a concomitant increase in adipocytes.^4^ Indeed, an increase in marrow adipocytes is observed in all conditions that lead to bone loss, such as aging,^5^ immobilization,^6^ microgravity,^7^ ovariectomy,^8^ anorexia nervosa,^9^ and treatment with glucocoticoids.^10^, ^11^ Although the pathogenesis of osteoporosis is multifactorial, these observations suggest that differentiation of BMSCs into adipocytes at the expense of osteoblasts is a major mechanism underlying osteoporotic disease.

The administration of steroid hormones such as glucocorticoids (GCs) is an effective and much-used anti-inflammatory therapy for several serious chronic diseases (eg, asthma and rheumatoid arthritis) and for preventing transplant rejection. GCs interact with the cognate intracellular glucocorticoid receptor (GR), which belongs to the nuclear receptor superfamily and regulates the transcription of a range of target genes. Hormone binding induces GR activation and translocation to the nucleus,^12^ where the hormone-receptor complex recognizes specific DNA sequences known as *glucocorticoid response elements* (GREs).^13^ In addition, GR also can modulate the expression of genes through a GRE-independent mechanism, such as protein-protein interaction of GR with other regulatory factors. Indeed, the main immunosuppressive and anti-inflammatory actions of GCs are mediated by *trans*-repression of the transcription factors AP-1 and nuclear factor-κB (NF-κB), which are known to regulate many genes involved in the immune response.^14–16^

Despite their considerable efficacy as anti-inflammatory agents, severe side effects frequently limit GC use. Among these is the development of osteoporosis,^17^ in which prolonged GC administration correlates with extended osteoclast life span, decreased osteoblast precursor proliferation and terminal differentiation, and increased apoptosis of both osteoblasts and mature osteocytes. Thus therapeutically administered GCs simultaneously reduce bone formation while enhancing reabsorption of bone tissue, generating an imbalance in the remodeling process that weakens the bone significantly.

Somewhat at odds with these observations in vivo, GCs are required in vitro for standard protocols of hBMSC differentiation to both osteoblasts and adipocytes. The work presented here investigates this apparent paradox, focusing on the molecular signals that regulate hBMSC proliferation with respect to osteogenesis and adipogenesis. We demonstrate that high concentrations of GC permit adipogenesis but impair osteogenesis by inhibiting proliferation and c-Jun expression. In contrast, the growth factor platelet-derived growth factor (PDGF) increases c-Jun activity and antagonizes the proadipogenic effects of GC administration. Thus the characterisation of this c-Jun-centered regulatory network provides insight on the differential response of hBMSCs to GCs that underpins the shift from osteogenesis to adipogenesis associated with GC-induced osteoporosis.

## Materials and Methods

### Culture and differentiation of hBMSCs

Human bone marrow–derived BMSCs (hBMSCs) were obtained from Inbiobank Stem Cell Bank (http://www.inbiobank.org). Cadaveric bone marrow was harvested from brain-dead donors after informed consent and under the Spanish National Organization of Transplant [Organización Nacional de Transplantes (ONT)] supervision. Each donor sample was tested and found negative for HIV-1/2, hepatitis B and C, cytomegalovirus, and *Mycoplasma*. All cells were processed at Inbiobank following manufacturing procedures based on ISO9001:2000 under good manufacturing practices (GMPs). Generated hBMSCs display a typical CD13^+^, CD29^+^, CD73^+^, CD90^+^, CD105^+^, CD166^+^, CD146^+^, CD34^–^, CD45^–^, CD14^–^, CD19^–^, and CD31^–^ phenotype; a fibroblast-like morphology; and at least trilineage potential, including osteocyte, chondrocyte, and adipocyte generation (Supplemental Fig. 1). hBMSCs were cultured in low-glucose DMEM (Sigma, http://www.sigmaaldrich.com) supplemented with 10% FBS preselected for BMSC optimal growth. On reaching confluence, hBMSCs were trypsinized and seeded at a density of 1 × 10^3^ cells/cm^2^ to expand cell culture constituting a passage. Cells were obtained in passage number 3 from the Stem Cell Bank. All experiments were carried out with cultures of low passage (passages 4 to 7). Included in the adipogenic medium weere 1 µM dexamethasone, 500 µM 2-isobutyl-1-methylxantine (IBMX), and 200 µM indomethacin (Sigma). Included in the osteogenic medium were 50 µM ascorbic acid, 10 mM β-glycerophosphate, and 0.1 µM dexamethasone (Sigma). For osteogenic visualization, cells were fixed using 70% ethanol and stained in alizarin red S 2% for 10 minutes. Stained cells were washed twice with distilled water. For quantification of staining, the monolayer was transferred with 10% acetic acid, heated to 85°C for 10 minutes, and transferred to ice for additional 5 minutes. Ammonium hydroxide was added to neutralize the acid. Samples were read at 405 nm in an Ultrospec 2000 spectrophotometer. SP600125, RU-486 (Mifepristone), and PDGF-BB were purchased from Sigma and used at the corresponding concentrations.

### shRNA- and cDNA-transduced cells

shRNA and cDNA vectors were constructed by using standard cloning procedures. c-Jun and GR shRNA oligonucleotides were purchased from Sigma-Genosys (http://www.sigmaaldrich.com): c-Jun (Accession No. NM_002228, sequence 1553–1573)^18^ and glucocorticoid receptor (Accession No. NM_000176, sequence 2086–2114; http://www.origene.com). Oligonucleotides were annealed and cloned into the pSUPER plasmid carrying the Pol III–dependent H1 promoter. The H1-shRNA expression cassette then was excised and cloned into the lentiviral pLVTHM plasmid using EcoRI-ClaI sites (Addgene Plasmid 12247, http://www.addgene.org).^19^ c-Jun cDNA was purchased from imaGenes (Clone IRATp970B0488D6, http://www.imagenes-bio.de), related to the NCBI c-Jun sequence NM_000228. c-Jun cDNA was excised and cloned into the pSP72 vector (Promega Plasmid P2191, http://www.promega.com) using EcoRI and BamH1. EcoRV and PvuII were used to get c-Jun cDNA from pSP72, and it was cloned in the PmeI cassette of pWPI plasmid (Addgene Plasmid 12254). c-Jun cDNA as well as GR and c-Jun shRNA sequences were checked after cloning in pWPI and pLVTHM vectors to confirm 100% correspondence with the original sequences. Viral particles were produced in human embryonic kidney 293T cells (ATCC, http://www.atcc.org). Briefly, 293T cells were seeded in high-glucose DMEM containing 10% FBS. pRRE, pREV, pVSV-G, and the lentiviral vector pLVTHM or pWPI containing both the GFP reporter gene and the shRNA or cDNA sequences were transfected in the packaging cell line by calcium phosphate precipitation in the presence of 25 µM chloroquine (Sigma). Transductions were carried out at various multiplicity of infections (MOIs) in the presence of 8 µg/mL of polybrene (Sigma). In order to achieve 100% infection, MOIs 9 and10 were used (for osteogenic differentiation assay and Western blot). To achieve 50% infection, MOIs 3 and 4 were used (for adipogenic differentiation and proliferation assays).

### Expression analysis

shRNA knockdown was evaluated by Western blot. Transduced or control cells were counted, and the same numbers of cells were lysed directly in sample buffer. Proteins then were separated in 12.5% (w/v) SDS-PAGE and subsequently transferred onto polyvinylidene difluoride membrane (PVDF). Antibodies to c-Jun, glutamine synthetase, glucocorticoid receptor (BD Biosciences Pharmingen, http://www.bdbiosciences.com), phosphorylated P-c-Jun (Ser 63) (Cell Signaling Technology), P-JNK (Upstate Biotechnology, Inc.), and cyclin D1 and Sam 68 (Santa Cruz Biotechnology, Santa Cruz, CA, USA, http://www.scbt.com) were used. Secondary antibodies were sheep or donkey anti-mouse or anti-rabbit, respectively, IgG conjugated with horseradish peroxidase (GE Healthcare UK, Ltd., Amersham Biosciences UK, Ltd., http://www.gehealthcare.com/lifesciences). Chemiluminescence was determined using SupersignalR Westfemto Maximum Sensitivity Substrate (Termo Scientific, http://www.piercenet.com). For GR immunofluorescence, cells were fixed with 4% paraformaldehyde and permeabilized with 0.1% Triton X-100. Secondary antibody α-mouse IgG Alexa 488 was used (Invitrogen, http://www.invitrogen.com). The nuclei were counterstained with Hoechst 33258 (Sigma).

### Proliferation assays

Methylthiazolyldiphenyl-tetrazolium bromide (MTT, Sigma) cell proliferation assays were carried out as described elsewhere.^20^ Briefly, cells were seeded at low cell concentrations (500 to 1000 cells/cm^2^) and cultured with the corresponding medium. Cells were first treated with 2 mg/mL of MTT at 37°C for 3 hours. The dye then was extracted with isopropanol HCl 0.04 N, and the water-insoluble formazan was solubilized for 2 hours before measurement of absorbance (570 nm) using an Ultrospec 2000 spectrophotometer. Cells were seeded at these low concentrations and then treated as mentioned earlier. When conditions reached confluence, an MTT proliferation assay was performed.

### Flow cytometry

For cell cycle analysis, hBMSCs were seeded at 1500 cells/cm^2^, serum starved, and subsequently stimulated with the corresponding treatment for an additional 24 hours. Cells were trypsinized and washed twice with fluorescence-activated cell-sorting (FACS) buffer (PBS, 1% heat-inactivated FBS, 1% bovine serum albumin) and then incubated in FACS buffer containing 0.1% saponin and subsequently stained with 1 mg/mL propidium iodide (Sigma) for 1 hour at 4°C. For adipocyte quantification, cells were cultured for the indicated time in the presence of adipogenic medium, and the adipocyte number was assessed using Nile red as described previously.^20^

## Results

### RNAi-mediated GR suppression substantially attenuates hBMSC adipogenesis and osteogenesis

**Fig 1:**
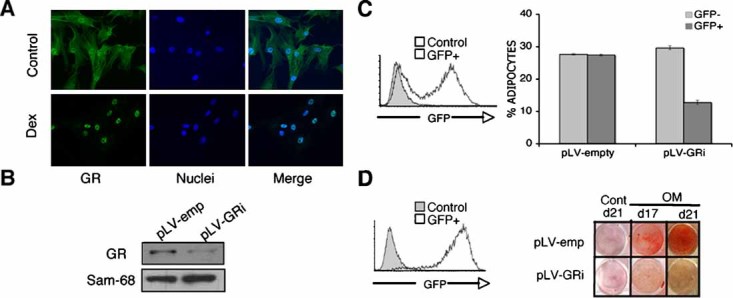
GR-specific gene silencing impaires osteo/adipogenesis capacity of hBMSCs. (*A*) Internalization of the glucocorticoid receptor in response to dexamethasone (Dex). hBMSCs were incubated in the presence of 1 µM Dex for 24 hours. (GR *green*, nuclei *blue*). (*B*) Western blot analysis of GR expression after specific knock-down in hBMSCs transduced with pLV-GRi. Control: pLVTHM empty vector (pLV-emp). Sam68 was used as a loading control. Effects of GR silencing on hBMSC adipogenesis (*C*) and osteogenesis (*D*). Analysis of GFP expression by flow cytometry. hBMSCs transduced with pLVTHM were harvested, and GFP expression was analyzed by flow cytometry (*white*). Negative control: noninfected hBMSCs (*gray*). hBMSCs transduced with either the empty or GRi-containing vector were induced to differentiate in the presence of adipogenic medium (AM) or osteogenic medium (OM). (*C*) Percentage of mature adipocytes stained with Nile red was estimated by flow cytometry. Percentage of GFP^+^ and GFP^–^ mature adipocytes after the induction period are represented by bars. Results show the mean ± SD of triplicate samples. Significance of the difference between GFP^+^ and GFP^–^ in pLV-GRi cells was determined using Student's *t* test; **p < .01. (*D*) Osteogenic induction of transduced hBMSCs. Cells were stained with alizarin red S. Control: absence of OM. Alizarin red calcium quantification is shown in Supplemental Fig. 2*A*.

We first confirmed that glucocorticoid receptor (GR) was responsive to steroid hormone in hBMSCs by incubating them with the synthetic glucocorticoid dexamethasone (Dex). As expected, Dex induced rapid translocation of GR into the nucleus (Fig. 1*A*). To clarify the role of this active GR in hBMSC differentiation, we used a GFP-expressing lentiviral vector (pLVTHM) to transduce hBMSCs at various multiplicities of infection with a stable GR-specific shRNA (GRi). This reduced GR protein expression to less than 10% of control (Fig. 1*B*). Using flow cytometry of Nile red staining and side scatter to quantify adipogenesis,^20^ we observed that cells in which the GR was “knocked down” (GFP^+^) generated almost threefold fewer adipocytes in adipogenic medium (AM) compared with pLV-empty GFP^+^ control cells (Fig. 1*C*). Inhibition of GR protein expression also blocked the synthesis of calcium matrix, as assessed by alizarin red S staining, in the presence of osteogenic medium (OM) (Fig. 1*D* and Supplemental Fig. 2*A*). This indicates that GR activity in hBMSCs is necessary for both osteogenesis and adipogenesis. To confirm that these GR-dependent effects were a consequence of GC interaction, we demonstrated that hBMSC differentiation in the presence of Dex to either an osteogenic or adipogenic fate was inhibited in a dose-dependent manner by RU-486, a GC antagonist used widely to examine GR-mediated Dex action (Supplemental Fig. 2*B*).

### GR signaling negatively regulates hBMSC proliferation

**Fig 2:**
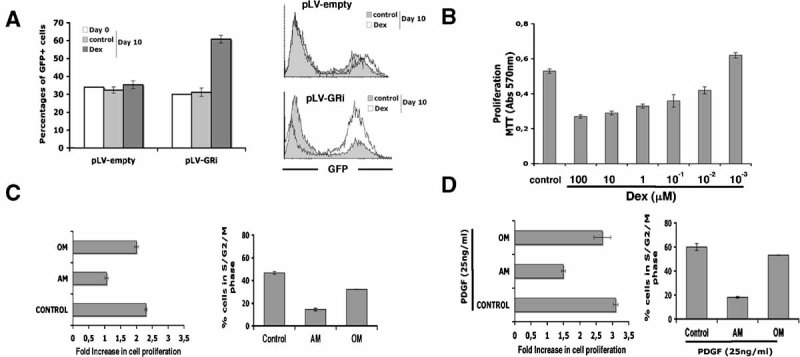
Effects of glucocorticoid receptor signaling on hBMSC proliferation. (*A*) hBMSC proliferation induced by specific GR inhibition in the presence of Dex. Transduced cells were plated at 500 cell/cm^2^ (day 0) in the presence or absence (control) of dexamethasone (1 µM) and analyzed by flow cytometry on day 10 (*left panel*). One representative experiment out of three is shown (*right pannel*). Significance of the difference between Dex-treated and control in pLV-GRi GFP^+^ cells was determined using Student's *t* test (*n* = 3, **p < .01). (*B*) hBMSC growth arrest induced by increasing concentrations of Dex. Noninfected hBMSCs were plated at 500 cell/cm^2^, and cell proliferation was estimated using the MTT assay on day 10 of culture. Significance was determined using Student's *t* test for paired samples relative to the proliferation levels measured on control cells (*n* = 3, **p < .01). (*C*) Effects of AM and OM on noninfected hBMSC cell growth. Noninfected hBMSCs were plated at 20000 cell/cm^2^ in the presence (AM or OM) or absence (control) of differentiation medium. Fold increase was estimated using the trypan blue dye exclusion assay and represents the cell count in relation to the cells plated on day 0. Cell cycle was analyzed by flow cytometry of hBMSCs stained with propidium iodide. The percentages of cells in S/G_2_/M are indicated. (*D*) hBMSCs were assayed under the same conditions as in panel *C* but in the presence of 25 ng/mL of PDGF. AM = adipogenic medium; OM = osteogenic medium. Results show the mean ± SD of triplicate samples. Significance of the difference between control and PDGF-treated cells from panels *C* and *D* is shown in Supplemental Fig. 3.

Although the differentiation process of hBMSCs toward the osteoblast/adipocyte lineages appears sensitive to GC, whether this effect can be dissociated from an effect on precursor proliferation is unclear. To investigate the role of GC/GR signaling on hBMSC proliferation, the ratio of GFP^+^ to GFP^–^ cells was determined after transduction of hBMSCs with the GFP-expressing GRi or control vectors. As expected, in the presence of Dex (1 µM), empty vector–transduced hBMSCs maintained a constant ratio of GFP expression throughout the culture period (10 days). In contrast, the percentage of GFP^+^ cells increased in the GRi-transduced cultures after reaching confluence (Fig. 2*A*). To further determine the effect on proliferative capacity, nontransduced hBMSCs (at 500 cells/cm^2^) were exposed to increasing concentrations of Dex (10^−3^ to 10^2^ µM), maintained in culture medium for 10 days, and assayed for cell proliferation in the presence of MTT. As shown in Fig. 2*B*, consistent with the previous observations, hBMSC proliferation was inhibited in a Dex concentration–dependent manner that peaked at 10 µM. More than 90% of the cells remained viable by trypan blue staining, excluding any deleterious effect of the synthetic hormone (data not shown). Thus these results confirm that GC/GR signaling negatively regulates hBMSC proliferation in a dose-dependent manner.

We have demonstrated previously that differentiation of hBMSCs into adipocytes requires inhibition of cell proliferation.^20^ However, the relationship between cell proliferation and the osteogenic potential of hBMSCs was unclear. To investigate, we observed that while expansion was negligible in AM medium (high Dex), hBMSCs (plated at 2 × 10^4^ cells/cm^2^) expanded 2- and 2.5-fold in OM (low Dex) or control medium, respectively (Fig. 2*C*). Cell cycle analysis by propidium iodide incorporation and subsequent flow cytometry for DNA content confirmed these results. As shown in Fig. 2*C*, AM-treated cells typically displayed only low levels of cells in S/G_2_/M (∼10%) compared with approximately 30% and approximately 40% cells in S/G_2_/M in OM and control medium, respectively. We have shown that several components of AM (including Dex) contribute to the negative effect on hBMSC proliferation.^20^ In contrast, Dex apart, the other components of OM (β-glycerol phosphate and ascorbic acid) did not affect hBMSC proliferation (data not shown). Thus our data collectively suggest that adipogenic and osteogenic differentiation of hBMSCs require a very different proliferation status that can be significantly influenced by GC/GR signaling activity.

### PDGF antagonizes GR-mediated effects on hBMSC proliferation and differentiation

**Fig 3:**
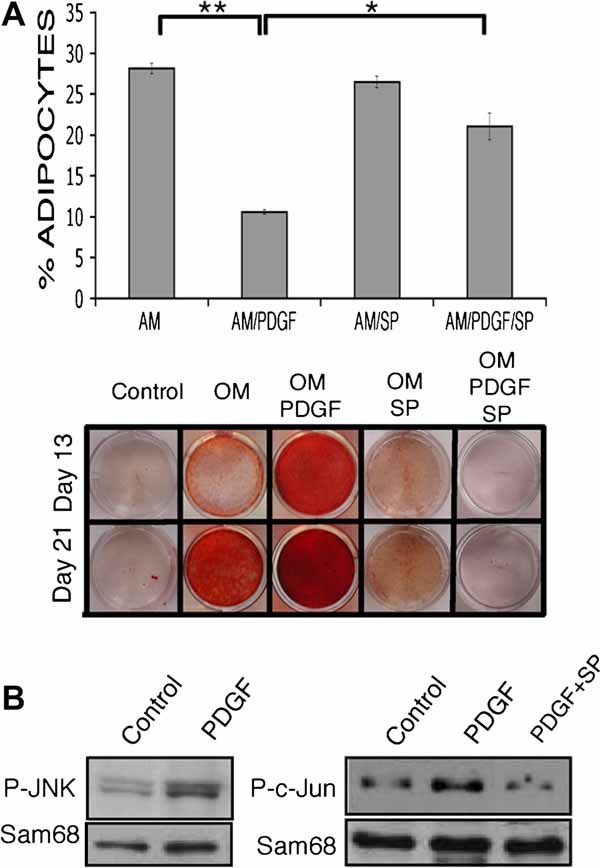
hBMSC differentiation capacity is influenced by the growth factor PDGF via JNK/c-JUN activation. (*A*) hBMSCs were induced to differentiate with AM or OM in the presence/absence of PDGF (25 ng/mL) and the specific JNK inhibitor SP600125 (4 µM). Percentages of mature adipocytes were determined by flow cytometry (*upper panel*). Results show the mean ± SD of triplicate samples. Significance of the difference between AM and AM/PDGF ± SP600125-treated cells was determined using Student's *t* test; **p < .01, *p < .05. Mineralized bone structures were revealed with alizarin red S stain (*bottom panel*). (*B*) PDGF induced activation of JNK/c-Jun in hBMSCs. Cells were stimulated with PDGF (25 ng/mL), and JNK and c-Jun phosphorylation was determined by immunoblot analysis using phosphospecific JNK and c-Jun antibodies. hBMSCs were pretreated for 6 hours with the inhibitor of JNK SP600125 (4 µM) or with the vehicle control, which significantly prevented the specific phosphorylation of c-Jun. Sam68 was used as a loading control.

Various growth factors, such as PDGF, have been shown to promote proliferation of hBMSCs.^20–23^ Since growth and differentiation are tightly linked processes, we questioned how proliferation affected the adipogenic and osteogenic capacity of hBMSCs. As shown in Fig. 2*D* and Supplemental Fig. 3, PDGF partially rescues the inhibition of proliferation that is observed under both AM and OM conditions, as measured by fold expansion and percentage of cells in the S/G_2_/M phases of the cell cycle. To investigate further, hBMSCs were induced to differentiate to both lineages in the presence or absence of PDGF. PDGF produced a greater than 60% reduction in adipocyte formation after 21 days of culture (Fig. 3*A*). In contrast, PDGF increased osteoblast differentiation from hBMSCs as mineralized extracellular matrix were observed faster and reached higher levels on alizarin red S staining (Fig. 3*A*, *bottom panel*).

The c-Jun NH_2_-terminal kinase (JNK) and, consequently, the c-Jun protein are reported targets of PDGF signaling. Thus, to elucidate the potential mechanism by which PDGF produces opposing effects on the adipogenic and osteogenic differentiation of hBMSCs, the activation state of JNK and c-Jun in PDGF-treated cultures was determined by immunoblot analysis. PDGF treatment resulted in the phosphorylation of both JNK and c-Jun (Fig. 3*B*). The PDGF-induced phosphorylation of c-Jun was completely prevented by pretreatment with the JNK inhibitor SP600125 (Fig. 3*B*). Furthermore, under AM conditions, SP600125 was able to rescue the PDGF-mediated reduction in the percentage of hBMSCs that differentiated into adipocytes (Fig. 3*A*, *upper panel*). In contrast, the osteogenic potential of hBMSCs was reduced in the presence of SP600125, even when PDGF was added (Fig. 3*A*, *bottom panel*). Taken together, these data implicate JNK/c-Jun activation in the opposing actions of PDGF on the adipogenic and osteogenic capacity of hBMSCs.

### GR and PDGF oppositely regulate c-Jun protein expression in hBMSCs

**Fig 4:**
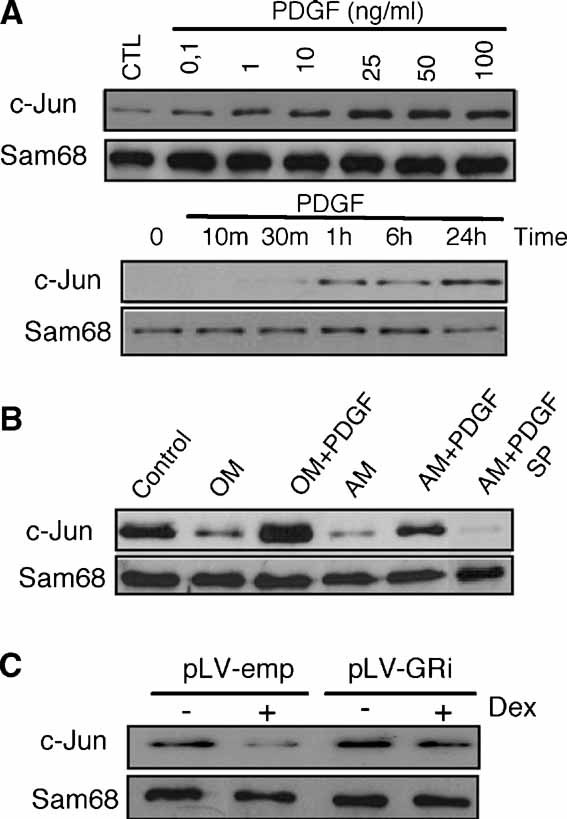
Glucocorticoid receptor activation downregulates c-Jun protein expression level and antagonizes the PDGF-induced signaling pathway. (*A*) Time- and dose-dependent effects of PDGF on c-Jun protein expression level by Western blot analysis. hBMSCs were treated with the indicated concentrations of PDGF for 24 hours (*upper panel*) or with either 25 ng/mL of PDGF or vehicle control (CTL) for the indicated time (*bottom panel*). (*B*) AM- or OM-induced downregulation of c-Jun expression level that antagonizes PDGF-induced upregulation of c-Jun via the JNK signaling pathway. hBMSCs were exposed to AM or OM in the presence of PDGF (25 ng/mL) or vehicle control for 24 hours. c-Jun expression level was measured by Western blot in total cell extracts using the antibody for c-Jun. hBMSCs were pretreated for 6 hours with the inhibitor of JNK SP600125 (4 µM), which significantly inhibited PDGF-induced c-Jun expression in the presence of AM. (*C*) Downregulation of c-Jun protein expression requires GR activation by dexamethasone. c-Jun protein levels were analyzed in Dex-treated (1 µM, 24 hours) hBMSCs transduced with either the empty or GRi-containing vector, as described in Fig. 1. Sam68 was used as a loading control. Protein quantification is shown is Supplemental Fig. 6*B*.

c-Jun plays a role in many cellular processes in response to growth factor ligands, forming Jun-Jun and Fos-Jun dimmers and activating AP-1 sites. Curiously, among important regulatory elements identified in the c-Jun promoter are two AP-1 sites, which suggest that c-Jun transcription could be regulated by its own gene product.^24^, ^25^ To further understand the role of c-Jun in the regulation of hBMSC proliferation and differentiation, c-Jun expression kinetics were investigated after PDGF treatment. PDGF elicited a rapid induction of c-Jun protein expression in a dose-dependent manner that peaked at 25 ng/mL (Fig. 4*A*, *top panel*). At this concentration of PDGF, c-Jun was first evident at 30 minutes and peaked at 24 hours (Fig. 4*A*, *bottom panel*), suggesting that in hBMSCs, c-Jun phosphorylation and subsequent activation stimulate its own transcription.

We next determined whether AM or OM conditions affected c-Jun expression in hBMSCs. Both media resulted in a considerable reduction in c-Jun protein at 24 hours by Western blot (Fig. 4*B*). Interestingly, a similar inhibition also was observed for cyclin D1, a proliferation-inducing gene known to be AP-1-responsive^26^ (Supplemental Fig. 4). Analysis of individual components of the differentiation medium showed that Dex was the major factor that reduced c-Jun protein levels and that the higher Dex concentration in AM versus OM was responsible for the different c-Jun levels observed between the two treatments (Supplemental Fig. 5). Significantly, downregulation of c-Jun expression by AM or OM was reversed in the presence of PDGF (Fig. 4*B*). This rescue depended on PDGF-mediated JNK signaling because SP600125 treatment abolished the induction of c-Jun expression (Fig. 4*B*).

Since Dex appeared to reduce c-Jun expression when hBMSCs were cultured in OM or AM, we next investigated the role of GR in c-Jun regulation by using the GRi vector. In GR knockdown conditions, Dex did not decrease cyclin D1 protein expression (Supplemental Fig. 6*A*), whereas c-Jun protein levels were significantly maintained (Fig. 4*C* and Supplemental Fig. 6*B*). Thus these data suggest that both activated GR and PDGF could modify hBMSC differentiation through their effects on c-Jun expression and subsequent cellular proliferation.

### Modulation of c-Jun expression regulates hBMSC proliferation and differentiation

**Fig 5:**
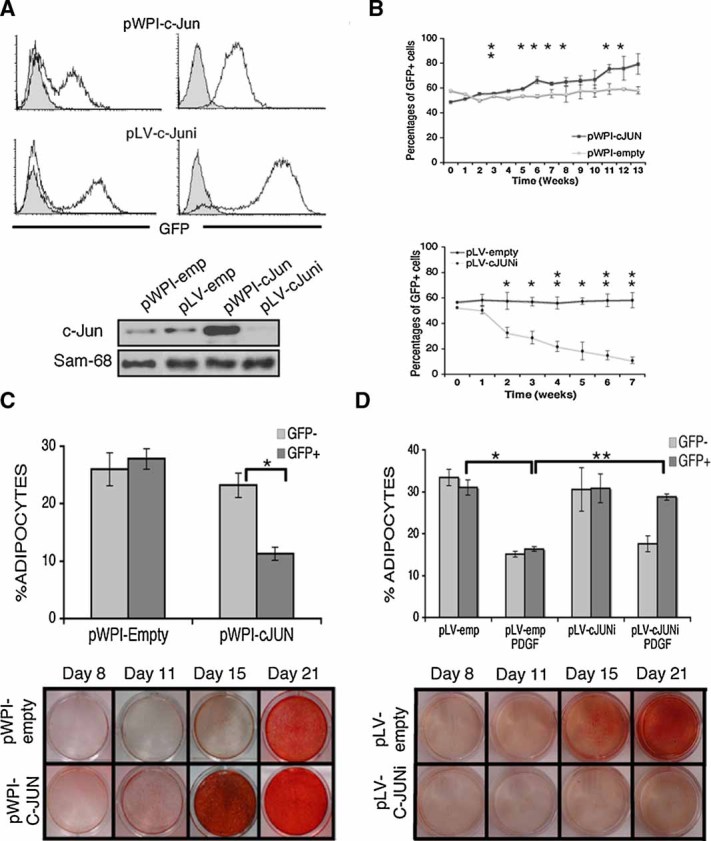
c-Jun expression level is involved in hBMSC proliferation and influences hBMSC differentiation capacity. (*A*) hBMSCs were transduced at different MOIs with the bicistronic pWPI-c-Jun cDNA or pLV-c-Juni RNAi expression vector, and GFP expression was analyzed by flow cytometry. Western blot analysis of c-Jun expression after specific knockdown or overexpression. Transduced cells (100% infected) were analyzed for protein expression by Western blot using a specific c-Jun antibody. Control: hBMSCs transduced with the empty vector (pLV-emp). Sam68 was used as a loading control. Protein quantification is shown in Supplemental Fig. 7. (*B*) c-Jun expression regulates hBMSC proliferation. hBMSCs showing approximately 50% infection efficiency (with either pWPI-c-Jun or pLV-c-Juni vector) were plated at 2000 cells/cm^2^, analyzed for GFP expression weekly, and replated at the same cell concentration until the end of the experiment. Control: cells transduced with the specific empty vector (pWPI or pLVTHM empty vector). Results show the mean ± SD of triplicate samples. pWPI-c-Jun and pLV-c-Juni were significantly different from the empty vector at all indicated time points. **p < .01, *p < .05. (*C*) c-Jun overexpression inhibits adipogenic capacity of hBMSCs. Percentage of mature adipocytes from GFP^–^ and GFP^+^ cells (cells transduced with the empty and c-Jun-cDNA expressing vectors, respectively) after the induction period are represented. c-Jun overexpression stimulates the osteogenic capacity of hBMSCs. Formation of mineralized extracellular matrix in hBMSCs transduced with either the empty vector (pWPI-empty) or the vector expressing c-Jun cDNA (pWPI-c-Jun), visualized by alizarin red S staining, is shown (*lower panel*). Significance of the difference between GFP^+^ and GFP^–^ in pWPI-c-Jun cells was determined using Student's *t* test; *p < .05. (*D*) c-Jun knockdown rescues adipogenesis from the inhibition caused by PDGF. hBMSCs transduced with either the empty (pLV-emp) or c-Juni-expressing vector (pLV-c-Juni) were induced to differentiate to adipogenic lineages in the presence or absence of PDGF (25 ng/mL). c-Jun knockdown inhibits hBMSC osteogenic differentiation. hBMSCs transduced with the pLV-c-Juni vector reduced the quantity of mineralized extracellular matrix extensively compared with control cells (pLV-empty). Results show the mean ± SD of triplicate samples. Significance of the difference between pLV-empty GFP^+^ cells in the presence/absence of PDGF (*p < .05) and in the presence or absence of c-Jun (**p < .01) was determined using Student's *t* test.

To further explore the role of c-Jun in hBMSC differentiation, we used our GFP-expressing lentiviral vectors to transduce approximately 50% of cultures of hBMSCs with either c-Jun cDNA (pWPI-c-Jun) or c-Jun-specific shRNA (pLV-c-Juni) or with empty-vector controls (Fig. 5*A*). The pWPI-c-Jun vector increased c-Jun protein more than 4-fold, whereas pLV-c-Juni reduced c-Jun protein to less than 5% of control (Fig. 5*A* and Supplemental Fig. 7). Since adipogenic and osteogenic potential of hBMSCs is related to cell cycle progression, we first determined whether c-Jun expression levels had any effect on hBMSC proliferation by analysis of the GFP^+^ to GFP^–^ ratio in hBMSCs transduced (∼50%) with the empty, c-Jun cDNA, or c-Juni viral vectors. As expected, hBMSCs infected with both empty vectors maintained a constant ratio of GFP expression throughout the culture period (Fig. 5*B*). In contrast, c-Jun overexpression produced a continuous, albeit small, increase in transduced cells from week 6 of the culture, whereas cells transduced with shRNA c-Juni decreased in number rapidly after week 2 to reach less than 10% of the culture at week 7 (Fig. 5*B*). Analysis of spontaneous cell death using Anexin-V revealed no differences in basal apoptotic rates between GFP^+^ and GFP^–^ cells (data not shown). Collectively, these data suggest that regulation of c-Jun expression is critically important for hBMSC proliferation.

We next tested hBMSC differentiation in AM or OM conditions after modulating c-Jun levels with vectors carrying either c-Jun cDNA or c-Jun-specific shRNA. In AM, pWPI-c-Jun-transduced cells (GFP^+^), which overexpressed c-Jun, produced less than 50% of the adipocytes generated by nontransduced cells (GFP^–^) (Fig. 5*C*). In contrast, pLV-c-Juni-transduced cells, in which c-Jun levels were reduced, maintained a comparable level of adipogenesis as nontransduced control cells in the same cultures (Fig. 5*D*). Furthermore, knockdown of c-Jun rescued the defect in adipocyte generation observed on culture with PDGF to almost normal levels (Fig. 5*D*). Manipulation of c-Jun had an opposite effect on the osteogenic differentiation of hBMSCs. c-Jun overexpression resulted in areas of mineralized extracellular matrix appearing more quickly and achieving higher levels than control cultures (Fig. 5*C*, *bottom panel*). Conversely, the reduction of c-Jun expression produced a complete inhibition of the synthesis of mineralized extracellular matrix (Fig. 5*D*). Taken together, these data demonstrate that c-Jun protein levels in differentiating hBMSCs are intimately linked to both proliferation and the balance between adipogenesis and osteogenesis.

### GR and AP-1 crosstalk determine the differentiation capacity of hBMSCs

**Fig 6:**
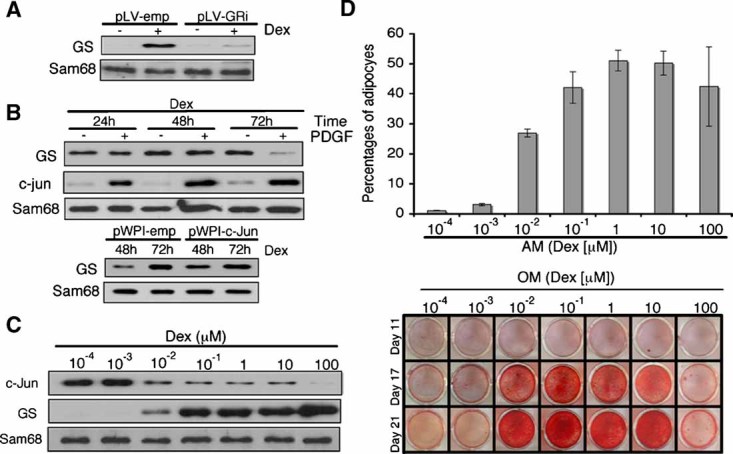
GR and c-Jun crosstalk determines the differentiation capacity of hBMSCs. (*A*) Western blot for glutamine synthetase (GS) on cell extracts from hBMSCs transduced with empty-vector or pLV-GRi and cultured in the presence or absence of Dex (1 µM) for 24 hours. (*B*) Regulation of c-Jun and GS expression by PDGF and Dex (*upper panel*). hBMSCs were cultured with Dex and in the presence or absence of PDGF (25 ng/mL) for the indicated time (*upper panel*). Membrane was sequentially immunoblotted with specific c-Jun and GS antibodies. GS expression analysis of c-Jun-overexpressing hBMSCs after Dex treatment (*bottom panel*). Cells transduced with either the empty or c-Jun cDNA-expressing vectors were cultured in the presence of Dex (1 µM) for the indicated time, and GS expression levels were determined by Western blot analysis. Sam68 was used as a loading control. (*C*) hBMSCs were incubated for 24 hours in the presence of the indicated concentrations of Dex. c-Jun and GS expression levels were determined by immunoblotting. (*D*) High concentrations of Dex impair osteogenesis but maintain adipogenic capacity in hBMSCs. Cells were induced to differentiate to adipocytes (*above*) or osteoblasts (*below*) for the indicated time in the presence of increasing concentrations of Dex. Percentages of mature adipocytes after the induction period are represented by bars. Results show the mean ± SD of triplicate samples. There is a significant difference between Dex concentrations in adipocyte induction, as tested by ANOVA (*p* < .001). Tukey's HSD post hoc analysis of these data yielded two homogeneous subsets, significantly different from each other at *p* < .05. The first subset includes concentrations ranging from 10^−4^ to 10^−3^ µM. The second subset includes concentrations above 10^−3^ µM, which induce higher percentages of adipocytes. So we conclude that treatment with Dex at 10^−2^ µM and above significantly enhances adipogenesis. Formation of mineralized extracellular matrix was visualized by alizarin red staining.

We next checked whether c-Jun protein levels interfered with GR activity in hBMSCs. Glutamine synthetase (GS) is a glucocorticoid-inducible enzyme that has an essential role in the metabolism of glutamine.^27^ Using a GS-specific antibody, we showed that GRi-expressing “knockdown” hBMSCs were unable to express GS protein in the presence of Dex (Fig. 6*A*). To evaluate whether c-Jun expression had any effect on GS expression, we added PDGF in the presence of Dex. Although we observed c-Jun induction in the presence of PDGF, GS accumulation was largely unchanged at 24 and 48 hours, only showing a significant decrease at 72 hours (Fig. 6*B*, *upper panel*). However, this PDGF-induced inhibition of GS levels was not dependant on c-Jun, because overexpression of c-Jun in hBMSCs (via pWPI-c-Jun) did not affect GS accumulation (Fig. 6*B*, *bottom panel*). This suggests that PDGF uses a c-Jun-independent pathway to interfere with GR activity.

To investigate the reciprocal relationship, we evaluated the effects of different concentrations of Dex on c-Jun and GS expression levels in 24-hour cultures of hBMSCs. Dex produced a concentration-dependent decrease in c-Jun that was correlated with increased GS expression (Fig. 6*C*). Nonetheless, there was a concentration window of Dex (10^−2^ to 10 µM) where both GR and c-Jun appear to be active (Fig. 6*C*).

Finally, we investigated whether there was an effect of increasing concentrations of Dex (10^−3^ to 10^2^ µM) on the adipogenic and osteogenic differentiation capacity of hBMSCs. Commonly used Dex concentrations (0.1 and 1 µM) generated both osteoblasts and adipocytes (Fig. 6*D*). Half-normal percentages of adipocytes were generated at 10^−2^ µM, with almost residual differentiation below that concentration. In contrast, areas of mineralized extracellular matrix were observed at concentrations as low as 10^−2^ µM (Fig. 6*D*). Below this concentration, we were not able to detect any osteoblast differentiation. Interestingly, an opposite effect was seen when higher concentrations of Dex were used; treatment with 10^2^ µM Dex resulted in a decrease in areas of mineralized extracellular matrix, whereas the adipogenic capacity of hBMSCs was maintained (Fig. 6*D*). Taken together, our data demonstrate that there is an optimal concentration of Dex where both osteocyte and adipocyte lineages can be generated. This coincides with GR-mediated induction of gene expression (eg, GS synthesis) and *trans*-repression of AP-1 (c-Jun downregulation) to levels that allow adipogenesis. Maximal GR-mediated *trans*-repression by higher Dex concentrations reveals a threshold of c-Jun downregulation that inhibits osteoblast generation while adipogenic capacity is maintained.

## Discussion

Normal skeletal maintenance requires a balance between mature osteoclasts and osteoblasts that implement a bone-remodeling process. This process is sensitive to glucocorticoid treatment, resulting in a rapid decline in bone mass on prolonged administration.^28^ In this report we have studied the effect of the synthetic glucocorticoid Dex on the differentiation of human bone marrow–derived BMSCs that represent a common precursor for both osteoblasts and adipocytes. Indeed, these results have allowed us to propose a new model in which crosstalk between c-Jun and the glucocorticoid receptor (GR) regulates the osteogenic and adipogenic potential of hBMSCs (Supplemental Fig. 8).

Glucocorticoid signaling plays a central role in regulating hBMSC activity. Indeed, inhibition of GR expression using an shRNA demonstrates a necessary role for GC-GR interactions for both osteogenesis and adipogenesis. In addition, increasing concentrations of Dex reduce hBMSC proliferation in a dose-dependent manner, an effect reversible by knockdown of GR levels. This notwithstanding, our data also demonstrate that osteogenesis and adipogenesis are differentially affected by GR signaling. Differentiation toward the adipogenic lineage, which requires the total absence of cell expansion, is possible at relatively high concentrations of Dex. In contrast, lower Dex concentrations are optimal for osteogenesis because differentiation to this fate is accompanied by hBMSC proliferation. This is consistent with the fact that adipogenic culture conditions (1 µM Dex) produce a profound block at the G_0_/G_1_ phase of the cell cycle compared with the almost normal hBMSC proliferation in osteogenic medium (0.1 µM Dex). Thus GR-regulated proliferation fundamentally affects hBMSC differentiation (Supplemental Fig. 8). In this context, previous models of bone cell differentiation invariably describe a phase of proliferation (followed by extracellular matrix maturation and mineralization).^29^ Thus proliferation may be required for the adoption of a certain gene expression program that subsequently supports the progressive acquisition of a fully differentiated osteogenic phenotype.

In contrast to glucocorticoid signaling, the PDGF pathway is known to promote hBMSC proliferation. Here we show that PDGF signaling also affects hBMSC differentiation. PDGF inhibits adipogenesis but serves to enhance osteogenesis, an observation consistent with the recent use of PDGF as a novel therapeutic tool to improve bone regeneration in clinical trials.^30^ We demonstrate that PDGF signaling in hBMSCs activates JNK, which, in turn, activates c-Jun, a component of the AP-1 complex that is implicated in many cellular processes, including proliferation, differentiation, and cellular transformation. Our data reveal that c-Jun overexpression recapitulates the effects of PDGF, conferring a slight proliferative advantage on hBMSCs while enhancing areas of mineralization in our osteogenic culture system. Consistent with this, c-Jun knockdown inhibited both hBMSC proliferation and osteogenesis. In contrast, elevated levels of c-Jun inhibited adipogenesis, whereas c-Jun knockdown rescued the PDGF-mediated reduction of adipocyte generation observed in AM culture conditions. This suggests that fine regulation of c-Jun levels, which also may include positive reinforcement of its own expression, is a critical parameter that controls lineage decisions between adipogenic and osteogenic fates (Supplemental Fig. 8). As we show here, c-Jun is known to upregulate expression of genes that induce proliferation, such as cyclin D1. In this context, it is interesting to note that c-Jun activity is increased in a variety of cancers, including high-grade osteosarcoma, where it seems critical for disease progression.^31^, ^32^ c-Jun is also highly amplified and overexpressed in human liposarcomas, leading to undifferentiated aggressive tumors associated with an early stage of adipocyte differentiation.^33^ Thus c-Jun amplification may confer an undifferentiated and more aggressive phenotype to an already developing tumor.

Our model, shown in Supplemental Fig. 8, proposes that crosstalk between glucocorticoid signaling through the glucocorticoid receptor and growth factor signaling (eg, PDGF) via the JNK/c-Jun pathway collaborate to dictate the differentiation potential of hBMSCs. Glucocorticoid administration to hBMSCs decreases c-Jun expression in a GR-dependent manner. This may occur by two main mechanisms: by direct protein-protein binding or by physical interaction with other cellular factors. Interestingly, the expression of c-Jun is rapidly and dramatically downregulated by glucocorticoids in many cell lines,^34^, ^35^ whereas several studies have documented interaction between AP-1 and activated GRs within the nucleus causing mutual repression of DNA binding.^36–38^ However, although our data from hBMSCs supports a direct role for GR in the regulation of c-Jun, PDGF-induced c-Jun does not appear to have a direct impact on GR signaling (as judged by GS expression levels). Instead, PDGF downregulates GR signaling with delayed kinetics (ie, 72 hours) independent of c-Jun levels.

Importantly, our results demonstrate that c-Jun expression and active GR signaling coexist at a range of glucocorticoid concentrations that permits hBMSCs to differentiate toward both the osteogenic and adipogenic lineages (Supplemental Fig. 8*A*). Elevation of c-Jun levels (eg, by PDGF; Supplemental Fig. 8*B*) and the consequent induction of proliferation (possibly via cyclin D1; Supplemental Fig. 9) interfere with adipogenesis, thus favoring osteogenesis (Supplemental Fig. 8*C*). Conversely, a reduction in active c-Jun, for example, when clinically administered Dex increases GR signaling, results in hBMSC growth arrest (Supplemental Fig. 10) and adipocyte differentiation at the expense of bone formation (Supplemental Fig. 8*D*).

Glucocorticoids are administered for a range of serious medical conditions, often in circumstances where no alternative therapies are available. A significant example is the use of glucocorticoids as immunosuppressant drugs, where the main immunosuppressive and anti-inflammatory actions of glucocorticoids are mediated by repression of AP-1 in cells of the immune system. Data presented here suggest that activation of the same pathway in hBMSCs could explain a serious side effect of glucocorticoid treatment, namely, bone fragility. It may prove problematic to separate unwanted glucocorticoid-mediated effects on AP-1 from those which generate anti-inflammatory responses. However, our study suggests that administration of agents such as PDGF, which increase c-Jun levels and temper GR signaling, predominantly in hBMSCs, may reduce these harmful side effects while maintaining efficacy in the immune system. These data also may improve our understanding of the mechanism of action of novel synthetic glucocorticoids that appear to have reduced side effects in recent clinical trials.^39^, ^40^ Indeed, an improved knowledge of the comparative effects of glucocorticoids in cells of different organ systems should pave the way for development of improved glucocorticoid-mediated therapies in the very near future.

## Conclusion

Glucocorticoids are used actively in the clinic as anti-inflammatory agents, but serious side effects, notably osteoporosis, limit their utility. This study aims at dissecting the role of GCs in mesenchymal stem cell proliferation and differentiation along the adipocyte versus osteoblast fate. Together our results indicate that higher concentrations of GC permit adipogenesis but impair osteogenesis, principally by inhibiting c-Jun expression and hBMSC proliferation. Conversely, osteogenesis is favored at concentrations of GC that support hBMSC proliferation but not adipogenic differentiation. We also demonstrate that the growth factor PDGF can antagonize the proadipogenic effects of GC administration by increasing c-Jun levels in a JNK-dependent manner. Indeed, our data have allowed us to propose a new model in which crosstalk between c-Jun and the glucocorticoid receptor regulates the differentiation potential of marrow hBMSCs.

## Disclosures

All the authors state that they have no conflicts of interest.
