# Supplementary figures and images for: Regulation of human bone marrow stromal cell proliferation and differentiation capacity by glucocorticoid receptor and AP-1 crosstalk
Source: J Bone Miner Res. 2010 Oct;25(10):2115–25. doi: 10.1002/jbmr.120 (PMC3607410; doi:10.1002/jbmr.120)

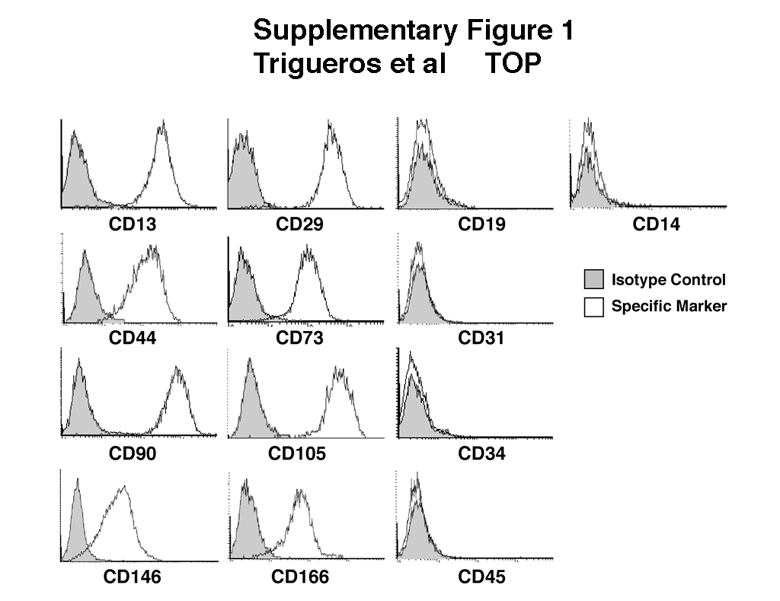

Supplement: Supplementary file 1 [file jbmr0025-2115-sd1.tif]

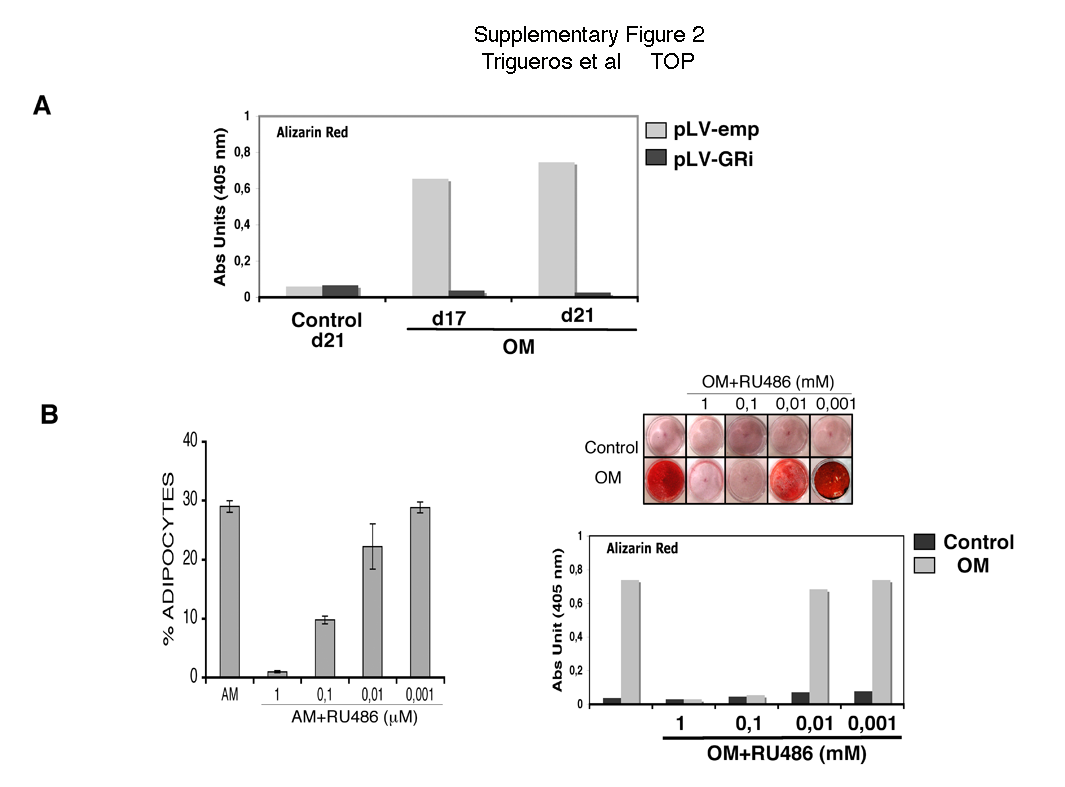

Supplement: Supplementary file 2 [file jbmr0025-2115-sd2.tif]

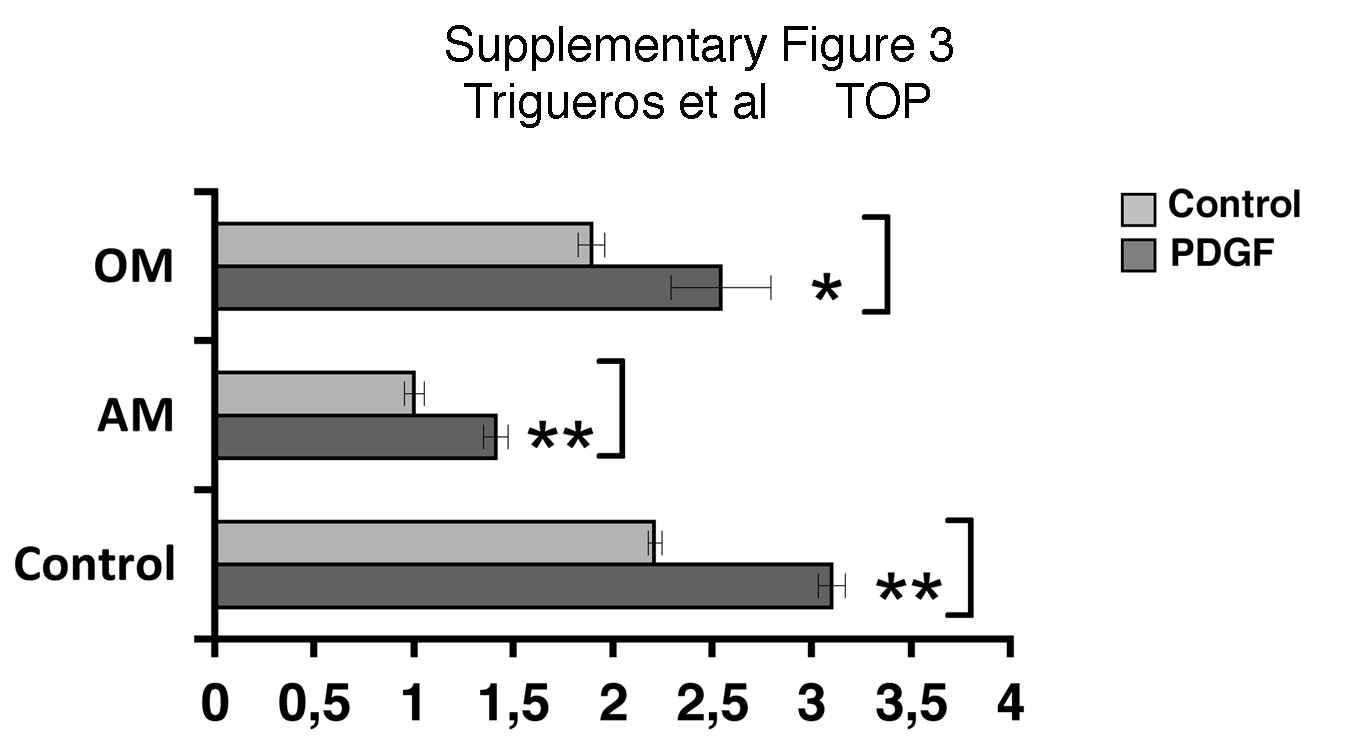

Supplement: Supplementary file 3 [file jbmr0025-2115-sd3.tif]

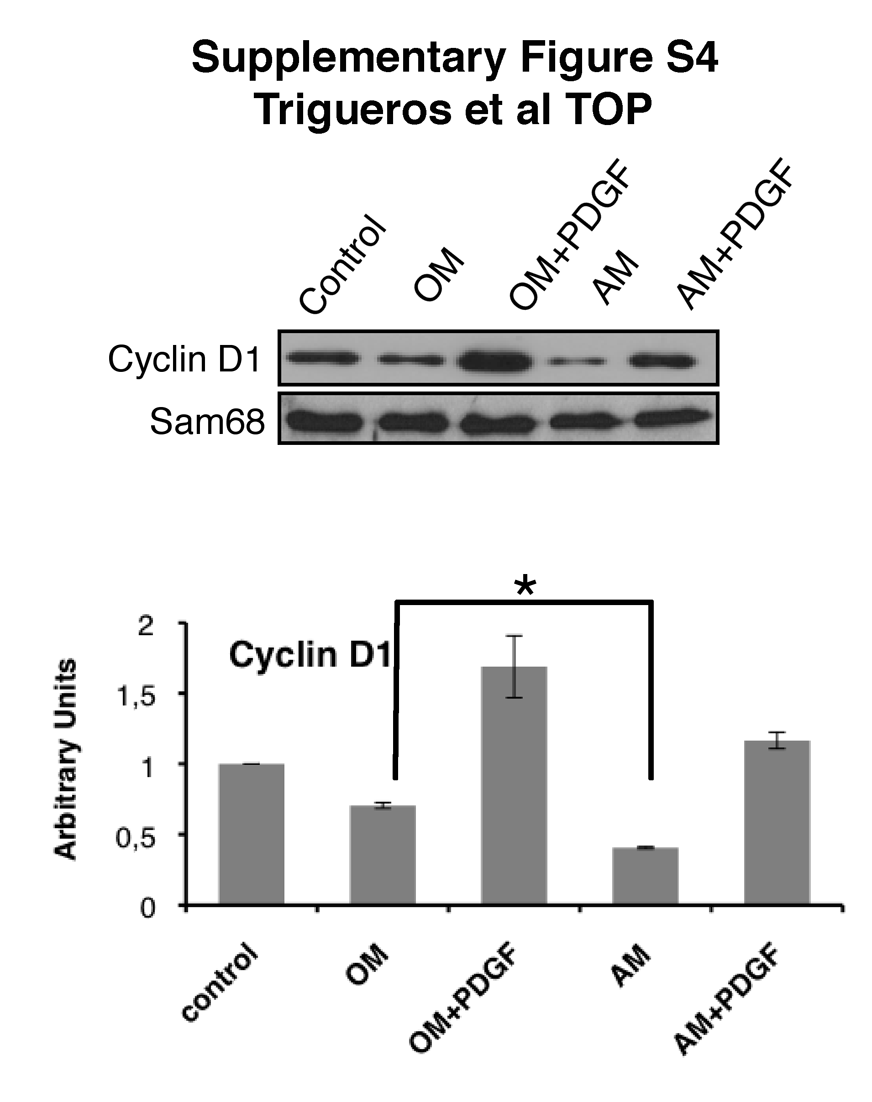

Supplement: Supplementary file 4 [file jbmr0025-2115-sd4.tif]

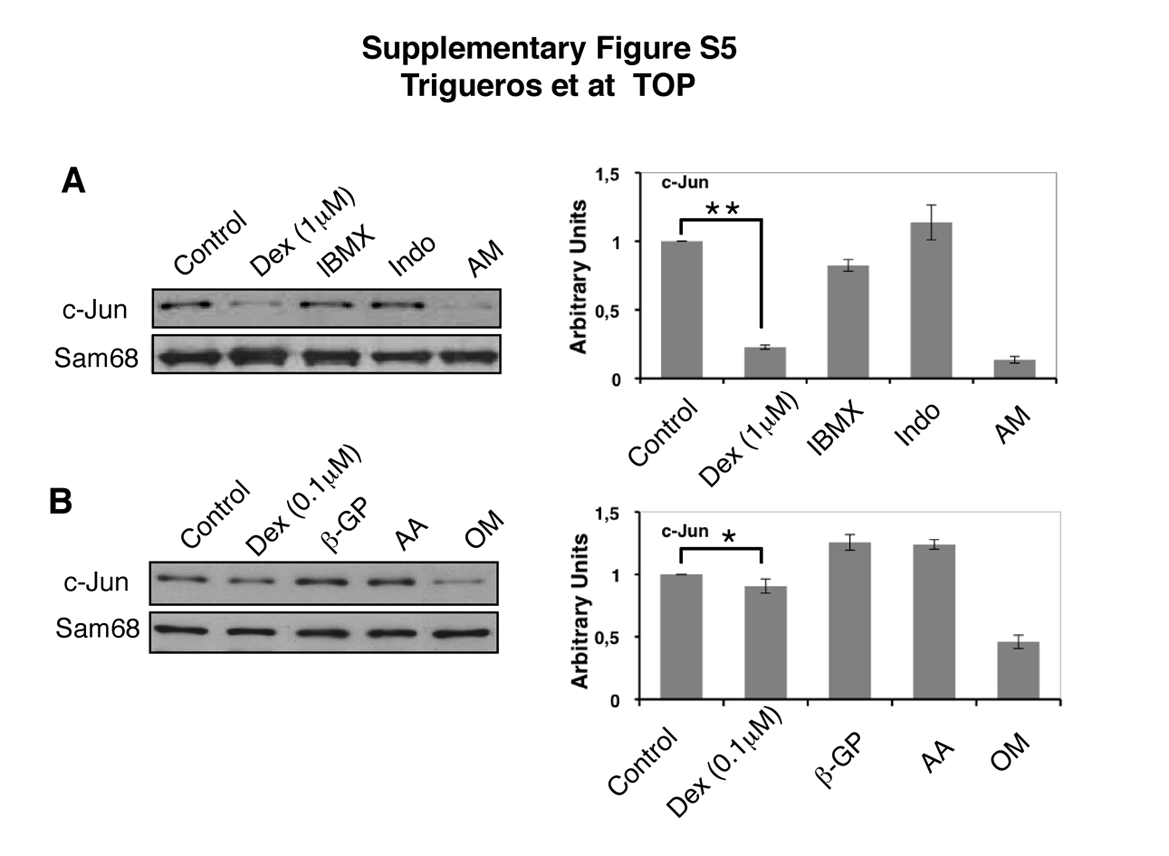

Supplement: Supplementary file 5 [file jbmr0025-2115-sd5.tif]

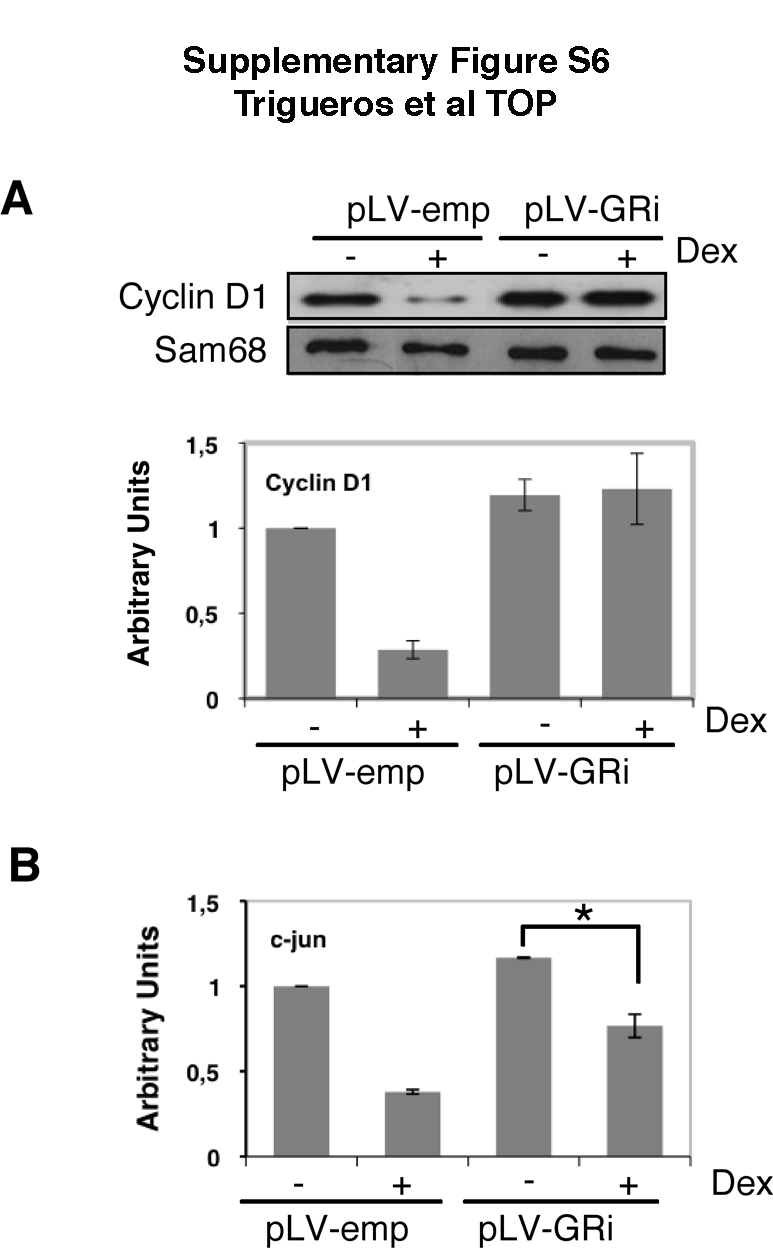

Supplement: Supplementary file 6 [file jbmr0025-2115-sd6.tif]

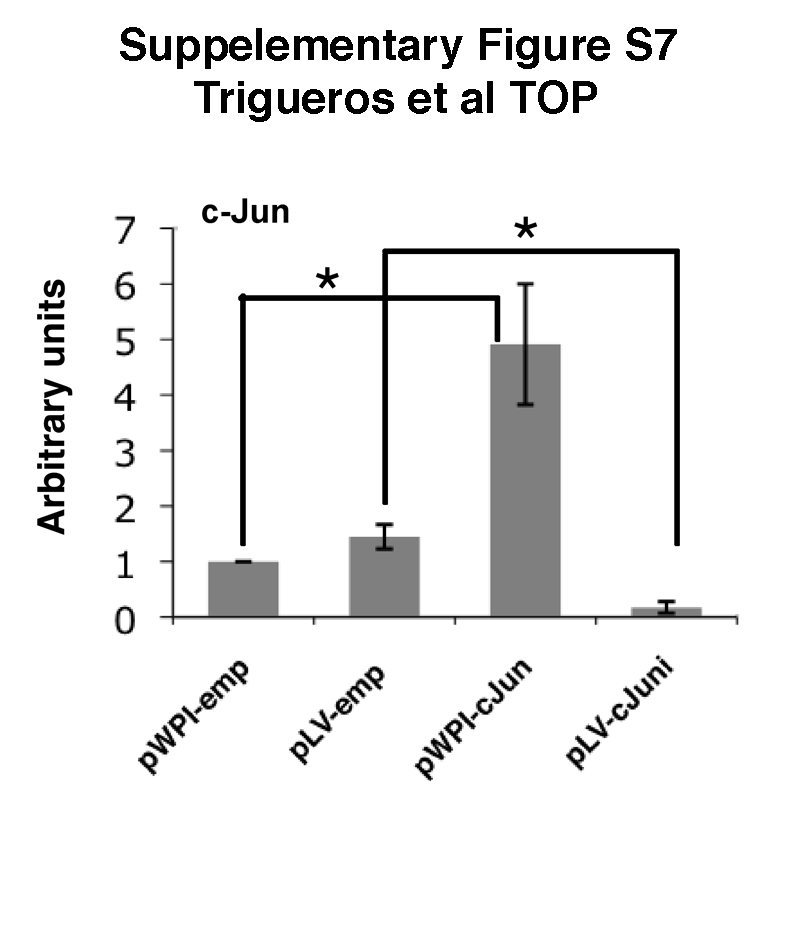

Supplement: Supplementary file 7 [file jbmr0025-2115-sd7.tif]

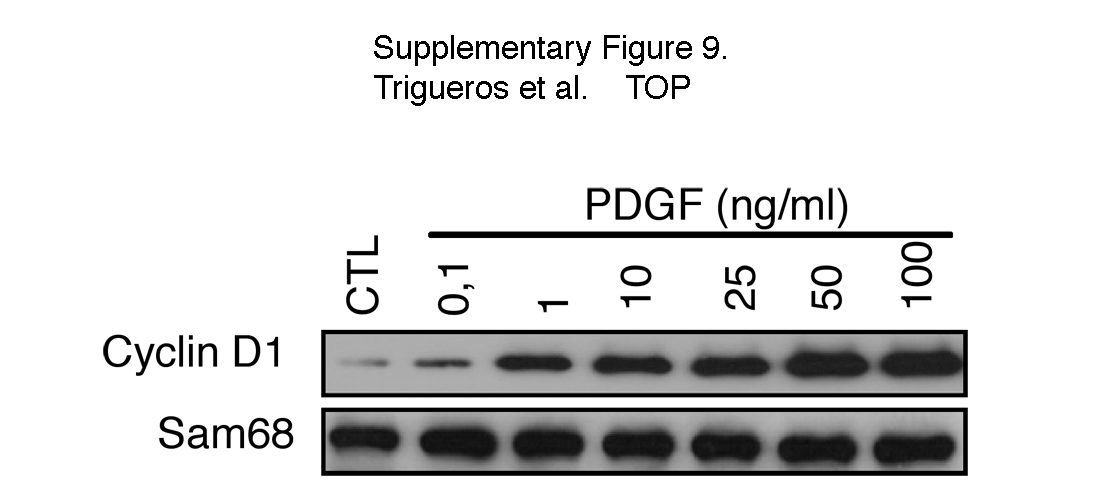

Supplement: Supplementary file 9 [file jbmr0025-2115-sd9.tif]

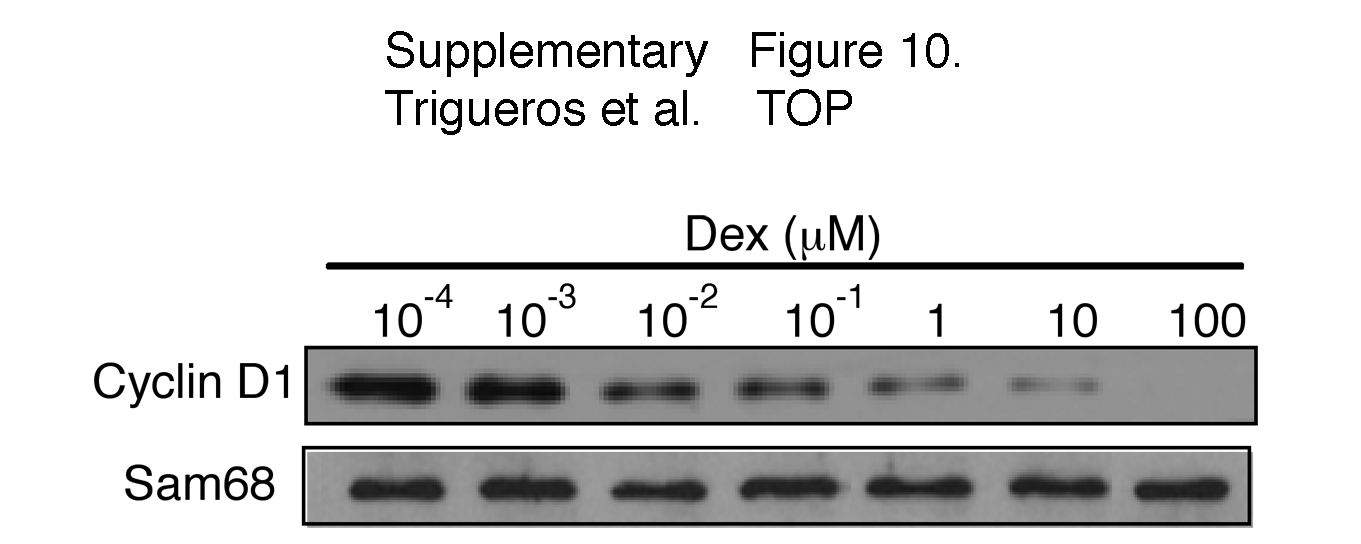

Supplement: Supplementary file 10 [file jbmr0025-2115-sd10.tif]
