# Supplementary material for: Regulation of human bone marrow stromal cell proliferation and differentiation capacity by glucocorticoid receptor and AP-1 crosstalk
Source: J Bone Miner Res. 2010 Oct;25(10):2115–25. doi: 10.1002/jbmr.120 (PMC3607410; doi:10.1002/jbmr.120)

Supplementary Figure 8
Trigueros et al. TOP

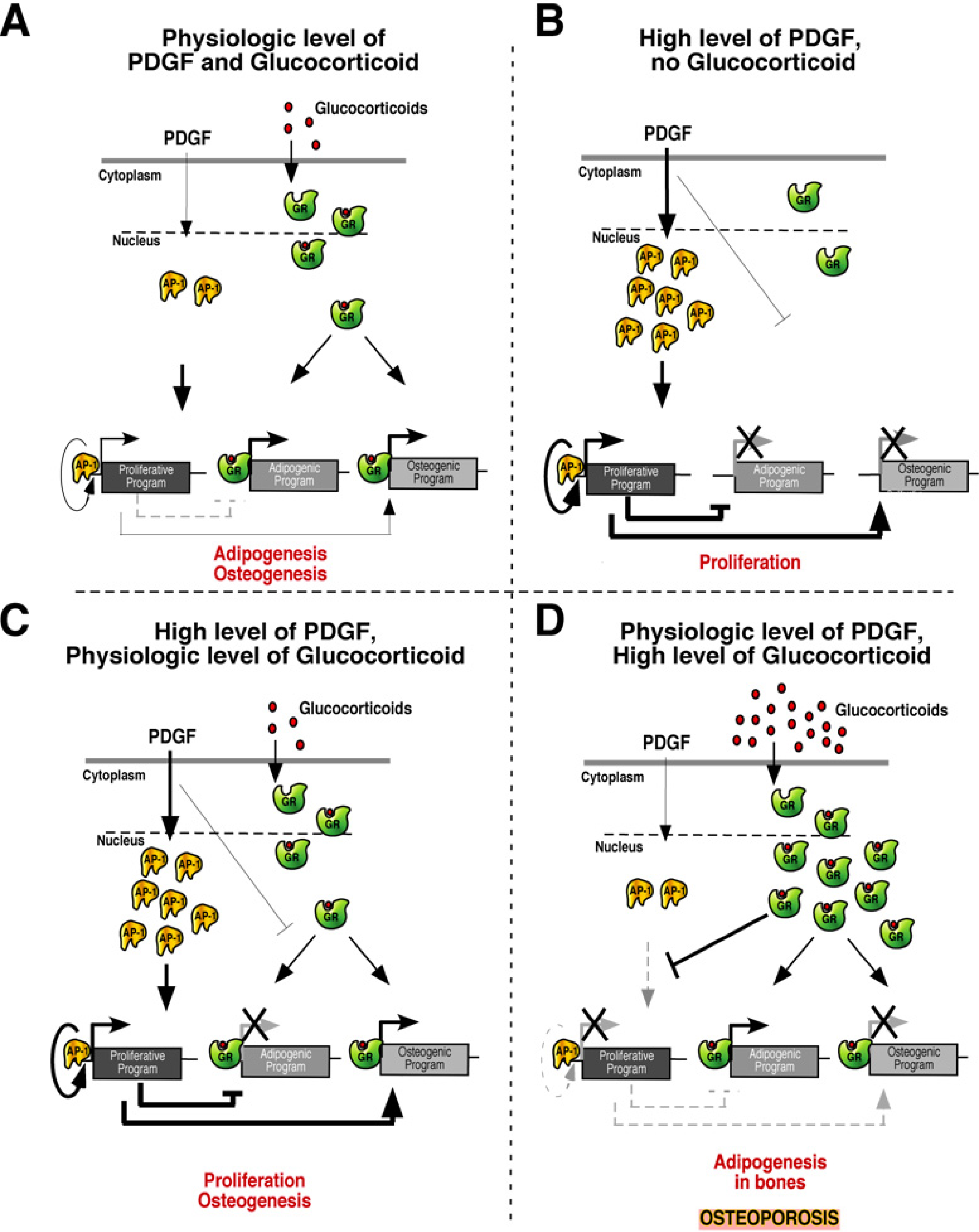

Supplement: Supplementary file 8 [file jbmr0025-2115-sd8.pdf]
